# Lumbar puncture for neurosyphilis investigation in asymptomatic patients with HIV-syphilis coinfection: a cross-sectional study among infectious disease specialists

**DOI:** 10.1590/1516-3180.2021.0744.R1.03032022

**Published:** 2022-08-29

**Authors:** Bárbara Labella Henriques, José Ernesto Vidal, Cristiano Gamba, Vivian Iida Avelino-Silva

**Affiliations:** I MD. Doctoral Student, Hospital das Clinicas HCFMUSP, Faculdade de Medicina, Universidade de Sao Paulo, Sao Paulo, SP, BR.; II MD. PhD. Infectious Disease Specialist, Department of Neurology, Instituto de Infectologia Emilio Ribas, São Paulo (SP), Brazil; Infectious Disease Specialist, Department of Infectious Diseases, Hospital das Clinicas HCFMUSP, Faculdade de Medicina, Universidade de Sao Paulo, Sao Paulo, SP, BR.; Universidade de Sao Paulo, Hospital das Clinicas HCFMUSP, Faculdade de Medicina, Department of Infectious Diseases, Sao Paulo, SP, Brazil; III MD. Infectious Disease Specialist, Centro de Referência e Treinamento DST AIDS (CRT), São Paulo (SP), Brazil.; IV MD, PhD. Infectious Disease Specialist, Department of Infectious Disease, Hospital das Clinicas HCFMUSP, Faculdade de Medicina, Universidade de Sao Paulo, Sao Paulo, SP, BR.

**Keywords:** Neurosyphilis, Syphilis, Infectious disease medicine, Cross-sectional studies, HIV, Cross-sectional survey, Human immunodeficiency virus, HIV-syphilis coinfection, Lumbar puncture

## Abstract

**BACKGROUND:**

Syphilis is a major public health issue worldwide. In people living with human immunodeficiency virus (PLHIV), there are higher incidences of both syphilis and neurosyphilis. The criteria for referring PLHIV with syphilis for lumbar puncture is controversial, and the diagnosis of neurosyphilis is challenging.

**OBJECTIVE:**

To describe the knowledge, attitudes, and practices of infectious disease specialists and residents in the context of care for asymptomatic HIV-syphilis coinfection using close-ended questions and case vignettes.

**DESIGN AND SETTING:**

Cross-sectional study conducted in three public health institutions in São Paulo (SP), Brazil.

**METHODS:**

In this cross-sectional study, we invited infectious disease specialists and residents at three academic healthcare institutions to answer a self-completion questionnaire available online or in paper form.

**RESULTS:**

Of 98 participants, only 23.5% provided answers that were in line with the current Brazilian recommendation. Most participants believed that the criteria for lumbar puncture should be extended for people living with HIV with low CD4^+^ cell counts (52.0%); in addition, participants also believed that late latent syphilis (29.6%) and Venereal Disease Research Laboratory (VDRL) titers ≥ 1:32 (22.4%) should be conditions for lumbar puncture in PLHIV with no neurologic symptoms.

**CONCLUSION:**

This study highlights heterogeneities in the clinical management of HIV-syphilis coinfection. Most infectious disease specialists still consider syphilis stage, VDRL titers and CD4^+^ cell counts as important parameters when deciding which patients need lumbar puncture for investigating neurosyphilis.

## INTRODUCTION

Syphilis is a major public health problem with increasing occurrence in several countries. In Brazil, data from the Ministry of Health show a three-fold increase in syphilis detection between 2014 and 2018, with incidence rates escalating from 25.1 to 75.8 cases per 100,000 person-years.^1^ Similar trends have also been reported in the United States, with a two-fold increase between 2014 and 2018,^2^ and in Europe, with greater risk among men who have sex with men.^3^

The prevalence of syphilis among people living with human immunodeficiency virus (PLHIV) is higher than in the general population. Studies performed in Brazil suggest that the prevalence of syphilis ranges from 2.7 to 20.5% among PLHIV;^4–6^ similarly, syphilis coinfection has been reported in 1%-21% of PLHIV in North America and 2%-43% in Europe.^7^

Besides its local manifestations, *Treponema pallidum* has systemic effects, notably, in the central nervous system. Conclusive diagnostic investigation of neurosyphilis may be challenging in the context of HIV coinfection, since serological and chemocytological abnormalities of the cerebrospinal fluid (CSF) may occur in PLHIV even without neurosyphilis. Moreover, given the high incidence of re-exposure to syphilis, the interpretation of the serological response after treatment may be challenging in this population.^8–10^

One of the most debated topics in the management of syphilis is the need and timing of CSF examination in HIV-syphilis coinfected patients with no neurologic symptoms. Guidelines and recommendations have been changing regarding this topic. Prior studies recommended a more aggressive approach with lumbar puncture based on CD4^+^ cell count, Venereal Disease Research Laboratory (VDRL) titers^11–13^ or syphilis stage.^14,15^ However, a less invasive approach suggests performing lumbar puncture based on criteria that are similar to those applied to HIV-uninfected individuals.^16,17^

As guidelines have been evolving and may present inconsistent recommendations, the clinical practice regarding investigation of asymptomatic neurosyphilis in PLHIV remains heterogenous. Cabana et al. argue that contradictory recommendations are an obstacle to effective adherence to guidelines.^18^ Other potential barriers include physicians’ lack of familiarity, agreement, or motivation for specific guidelines, favoring the persistence of previous practices. External factors including the inability to reconcile patient preferences, lack of time, lack of resources and organizational constraints also play a role in heterogenous practices.^18,19^

Based on our routine observation, we hypothesized that some providers may tailor decisions regarding lumbar puncture based on barriers faced to perform the exam (i.e., long waiting time, lack of trained practitioners, lack of an appropriate procedure room) or difficulties to implement neurosyphilis treatment after the diagnosis (i.e., absence of hospital service and long waiting time for hospitalization).

Few studies have investigated the knowledge and attitudes of healthcare providers regarding the management of syphilis-HIV coinfection,^20–24^ and studies exploring attitudes on the investigation of asymptomatic neurosyphilis in PLHIV are even more scarce.^25^

## OBJECTIVE

Our aim was to describe the knowledge, attitudes, and practices of infectious disease specialists in the context of asymptomatic HIV-syphilis coinfection using close-ended questions and case vignettes. We also explored if attitudes and practices of providers who report difficulties for lumbar puncture procedure and/or neurosyphilis in-hospital treatment varied among participants.

## METHODS

In this cross-sectional study, we invited infectious disease specialists and residents from three public and academic health-care institutions in São Paulo, Brazil, to answer a self-completion questionnaire. The institutions were selected based on the anticipated number of potential participants. Responses could be collected either in person (paper form) or online via a form sent to an institutional mailing list or through WhatsApp. The electronic form option was added due to the restrictions imposed by the coronavirus disease pandemic.

The questionnaire included demographic information, case vignettes of PLHIV with syphilis coinfection and no neurologic symptoms, and questions addressing knowledge about the clinical management of neurosyphilis in patients with HIV/syphilis coinfections based on the Ministry of Health recommendations in Brazil. We also investigated participants’ perceptions on barriers to refer patients to lumbar puncture or to neurosyphilis in-hospital treatment to explore if these aspects had any impact on questionnaire responses.

Demographics, training, and practice characteristics were collected in the first section of the questionnaire. Ten case vignettes with hypothetical situations addressing neurosyphilis investigation with lumbar punctures and interpretation of CSF laboratory reports were presented in the second part of the questionnaire. The final section explored the knowledge about the indications for lumbar puncture for neurosyphilis investigation in PLHIV according to recommendations in Brazil; criteria for lumbar puncture according to the participant's own opinion; and interpretation of CSF results.

Barriers for lumbar punctures and in-hospital neurosyphilis treatment were explored using ordinal close-ended responses. Participants were asked about the level of difficulty for a lumbar puncture in routine practice (not at all difficult; somewhat difficult; very difficult; cannot inform); and the level of difficulty in hospitalizing a patient with neurosyphilis for intravenous treatment with crystalline penicillin (not at all difficult; somewhat difficult; very difficult; cannot inform). To explore if participants’ perceptions on barriers to refer patients to lumbar puncture or to in-hospital treatment had any impact on questionnaire responses, we categorized study participants as: (i) Group 1: participants reporting no difficulties for lumbar puncture or patient hospitalization; and (ii) Group 2: participants reporting at least some difficulties for lumbar puncture and/or those who perceived patient hospitalization as very difficult.

The characteristics of the study participants were presented using frequencies and percentages for categorical variables and medians and interquartile ranges (IQR) for numeric variables. Comparisons between individual participants’ answers to case vignettes were performed using chi-squared tests or Fisher's exact tests, as appropriate. Two-tailed P < 0.05 were considered statistically significant for all the comparisons.

The data were inserted into the REDCap platform and analyzed using Stata 15.1 (StataCorp; StataCorp LP, College Station, Texas). Written informed consent was obtained from all participants, and no identifiable information was collected during the study.

### Ethical aspects

The study was approved by the Ethics Committee at the coordinating institution (Comissão de Ética para Análise de Projetos de Pesquisa – CAPPesq, Faculdade de Medicina da Universidade de São Paulo, CAAE: 19926919.1.0000.0068, July 20, 2020) and by the ethics committees at the collaborating institutions (Comitê de Ética, Instituto de Infectologia Emílio Ribas, CAAE: 19926919.1.3001.0061, July 22, 2020 and Comitê de Ética em Pesquisa, Centro de Referência DST/AIDS, CAAE: 19926919.1.3003.5375, September 10, 2020). All participants provided written or electronic informed consent. All individual identifiable information was maintained in secured cabinets and electronic files.

## RESULTS

### Participant characteristics

Between December 2019 and September 2020, 98 infectious disease specialists or residents responded to the survey. The demographics, training, and practice characteristics are described in Table 1. Ages ranged from 25 to 68 years (median 35.5 years old). Most participants (65.3%) were female, most (72.4%) had completed the Infectious Disease Residency Program, and 43 (43.9%) had a postgraduate degree. The vast majority (92.9%) reported providing medical care to PLHIV. Regarding professional activities, 76 participants (77.6%) declared working in a public hospital, 48 (49.0%) in a private hospital, 15 (15.3%) in a research project, 9 (9.2%) in intensive care units, and 8 (8.2%) in-hospital infection control programs (Table 1).

**Table 1:** Demographics, training, and practice characteristics of study participants, overall and according to group category

Characteristics		All participants n = 98	Group 1** n = 61	Group 2*** n = 37	P value
**Median age (IQR)**		34.5 (30-44)	36.0 (31-48)	33.0 (30-41)	0.138
**Female sex (%)**		64 (65.3)	43 (70.5)	21 (56.8)	0.193
**Time since graduation from medical school** *					
	< 5 years	18 (18.4)	12 (19.7)	6 (16.2)	
	5-10 years	31 (31.6)	15 (25.6)	14 (37.8)	
	11-20 years	22 (22.4)	13 (21.3)	9 (24.3)	0.477
	20-30 years	18 (18.4)	12 (19.7)	6 (16.2)	
	> 30 years	10 (10.2)	9 (14.7)	1 (2.7)	
**Infectious disease residency completed (%)**		71 (72.4)	45 (73.7)	26 (70.3)	0.816
**Master's/PhD ongoing or completed (%)**		43 (43.9)	28 (45.9)	15 (40.5)	0.677
**Provides medical care for PLHIV (%)**		91 (92.9)	56 (91.8)	35 (94.6)	0.707
**Professional activity (%)**					
	Public hospital	76 (77.6)	48 (78.7)	28 (75.7)	0.805
	Private hospital	48 (49.0)	29 (47.5)	19 (51.3)	0.835
	Clinical research	15 (15.3)	6 (9.8)	9 (24.3)	0.081
	Intensive care unit	9 (9.2)	5 (8.2)	4 (10.8)	0.726
	Hospital infant control program	8 (8.2)	4 (6.6)	4 (10.8)	0.471

* Missing for one participant; IQR, interquartile range;

** Group 1: Participants reporting no difficulties for lumbar puncture or patient hospitalization;

*** Group 2: Participants reporting at least some difficulties for lumbar puncture and/or those who perceived patient hospitalization as very difficult.

Regarding barriers for lumbar puncture and hospitalization, 27 (27.5%) of the study participants declared the perception that access to lumbar punctures is somewhat difficult, and 3 (3.1%) perceived access to lumbar punctures as very difficult; 56 (57.6%) perceived access to in-hospital treatment as somewhat difficult, while 14 (14.7%) declared that patient hospitalization for neurosyphilis treatment was very difficult. Group 2 included 37 participants who considered access to lumbar puncture somewhat difficult or very difficult and/or hospitalization very difficult.

Comparisons of the demographics, training, and practice characteristics according to group categorization are presented in Table 1.

### Responses to case vignettes

In the second section of the questionnaire, case vignettes with hypothetical situations addressing neurosyphilis investigation with lumbar punctures and interpretation of CSF laboratory reports were presented to participants, as described in Tables 2 and 3.

**Table 2:** Attitudes in case vignettes regarding neurosyphilis investigation with lumbar puncture among patients with HIV-syphilis coinfection with no neurologic symptoms

**Case 1: PLHIV diagnosed five years ago, under regular use of HAART, CD4^+^ cell count = 510 cells/mm³ and undetectable viral load. FTA-Abs reagent and VDRL 1:128 (FTA-Abs was negative in the previous test)** **1**				
	**Total n = 78**	**Group 1** ** **n = 48**	**Group 2** *** **n = 30**	**P value**
Expectant management and repeat VDRL in 3-6 months (%)	0 (0)	0 (0)	0 (0)	0.638
Treat with Penicillin G Benzathine and repeat VDRL in 3-6 months (%)#	60 (85.3)	35 (72.9)	25 (83.3)	
Refer to LP for neurosyphilis investigation (%)	17 (21.3)	12 (25.0)	5 (16.6)	
Another option (%)	1 (1.3)	1 (2.0)	0 (0)	
**Case 2: PLHIV diagnosed five years ago under regular use of HAART, CD4^+^ cell count = 110 cells/mm³ and undetectable viral load. FTA-Abs reagent and VDRL 1:128 (FTA-Abs was negative in the previous test)**				
	**Total n = 98**	**Group 1 n = 61**	**Group 2 n = 37**	**P value**
Expectant management and repeat VDRL in 3-6 months (%)	1 (1.1)	1 (1.6)	0 (0)	0.485
Treat with Penicillin G Benzathine and repeat VDRL in 3-6 months (%)#	33 (33.7)	18 (29.6)	15 (40.6)	
Refer to LP for neurosyphilis investigation (%)	64 (65.3)	42 (68.9)	22 (59.4)	
Another option (%)	0 (0)	0 (0)	0 (0)	
**Case 3: PLHIV diagnosed five years ago under regular use of HAART, CD4^+^ cell count = 510 cells/mm³ and undetectable viral load. FTA-Abs reagent and VDRL 1:128 (FTA-Abs was negative in the previous test). 12 months after treatment with Penicillin G Benzathine persists with VDRL = 1/32. Reports no reexposure** **2**				
	**Total n = 96**	**Group 1 n = 59**	**Group 2 n = 37**	**P value**
Expectant management and repeat VDRL in 3-6 months (%)	28 (29.2)	16 (27.1)	12 (32.4)	0.221
Treat with Penicillin G Benzathine and repeat VDRL in 3-6 months (%)	2 (2.1)	0 (0)	2 (5.4)	
Refer to LP for neurosyphilis investigation (%)#	65 (67.7)	42 (71.2)	23 (62.2)	
Another option (%)	1 (1.0)	1 (1.7)	0 (0)	
**Case 4: PLHIV diagnosed recently without HAART, CD4^+^ cell count = 110 cells/mm³ and viral load 112.900. FTA-Abs reagent and VDRL 1:4. Reports no previous treatment for syphilis** **3**				
	**Total n = 97**	**Group 1 n = 61**	**Group 2 n = 36**	**P value**
Expectant management and repeat VDRL in 3-6 months (%)	3 (3.1)	2 (3.3)	1 (2.8)	0.866
Treat with Penicillin G Benzathine and repeat VDRL in 3-6 months (%)#	43 (44.3)	25 (41.0)	18 (50.0)	
Refer to LP for neurosyphilis investigation (%)	50 (51.6)	33 (54.1)	17 (47.2)	
Another option (%)	1 (1.0)	1 (1.6)	0 (0)	
**Case 5: PLHIV diagnosed recently without HAART, CD4^+^ cell count = 430 cells/mm³ and viral load 112.900. FTA-Abs reagent and VDRL 1:32. Reports no previous treatment for syphilis** **3**				
	**Total n = 97**	**Group 1 n = 61**	**Group 2 n = 36**	**P value**
Expectant management and repeat VDRL in 3-6 months (%)	1 (1)	0 (0)	1 (2.8)	0.104
Treat with Penicillin G Benzathine and repeat VDRL in 3-6 months (%)#	54 (55)	30 (49.2)	24 (66.7)	
Refer to LP for neurosyphilis investigation (%)	40 (40.8)	29 (47.5)	11 (30.6)	
Another option (%)	2 (2)	2 (3.3)	0 (0)	

1 Missing for 20 participants (20.4%);

2 missing for two participants (2.1%);

3 missing for one participant (1.0%);

# Brazilian Guideline-recommended management; HAART, highly active antiretroviral treatment. PLHIV = people living with human immunodeficiency virus; HAART = Highly Active Antiretroviral Therapy; FTA-Abs = fluorescent treponemal antibody absorption; VDRL = Venereal Disease Research Laboratory; LP = lumbar puncture.

** Group 1: Participants reporting no difficulties for lumbar puncture or patient hospitalization;

*** Group 2: Participants reporting at least some difficulties for lumbar puncture and/or those who perceived patient hospitalization as very difficult.

**Table 3:** Attitudes in case vignettes regarding treatment of syphilis in patients with HIV-syphilis coinfection with no neurologic symptoms after lumbar puncture

**Case 1: PLHIV diagnosed five years ago under regular use of HAART, CD4^+^ cell count = 510 cells/mm³ and undetectable viral load. FTA-Abs reagent and VDRL 1:128. CSF: VDRL non-reagent, FTA-Abs reagent, 25 cells/mm^3^, protein 40 mg/dl** **1**				
	**Total n = 95**	**Group 1** ** **n = 59**	**Group 2** *** **n = 36**	**P value**
Neurosyphilis treatment: Intravenous Penicillin G/Ceftriaxone (%)	72 (75.8)	41 (69.5)	31 (86.1)	0.161
Syphilis treatment: Penicillin G Benzathine (%)	22 (23.3)	17 (28.8)	5 (13.9)	
Another option (%)	1 (1.1)	1 (1.7)	0 (0)	
**Case 2: PLHIV diagnosed five years ago under regular use of HAART, CD4^+^ cell count = 110 cells/mm³ and undetectable viral load. FTA-Abs reagent and VDRL 1:128. CSF: VDRL non-reagent, FTA-Abs reagent, 25 cells/mm^3^, protein 40mg/dl** **1**				
	**Total n = 95**	**Group 1 n = 59**	**Group 2 n = 36**	**P value**
Neurosyphilis treatment: Intravenous Penicillin G/Ceftriaxone (%)	84 (88.4)	50 (84.7)	34 (94.4)	0.378
Syphilis treatment: Penicillin G Benzathine (%)	10 (10.5)	8 (13.6)	2 (5.6)	
Another option (%)	1 (1.1)	1 (1.7)	0 (0)	
**Case 3: PLHIV diagnosed five years ago under regular use of HAART, CD4^+^ cell count = 510 cells/mm³ and undetectable viral load. FTA-Abs reagent and VDRL 1:128. CSF: VDRL non-reagent, FTA-Abs reagent, 8 cells/mm^3^, protein 55 mg/dl** **2**				
	**Total n = 97**	**Group 1 n = 60**	**Group 2 n = 37**	**P value**
Neurosyphilis treatment: Intravenous Penicillin G/Ceftriaxone (%)	56 (57.7)	33 (55.0)	23 (62.2)	0.384
Syphilis treatment: Penicillin G Benzathine (%)	37 (38.1)	23 (38.3)	14 (37.8)	
Another option (%)	4 (4.1)	4 (6.7)	0 (0)	
**Case 4: PLHIV diagnosed five years ago under regular use of HAART, CD4^+^ cell count = 110 cells/mm³ and undetectable viral load. FTA-Abs reagent and VDRL 1:128. CSF: VDRL non-reagent, FTA-Abs reagent, 8 cells/mm^3^, protein 55 mg/dl** **1**				
	**Total n = 95**	**Group 1 n = 59**	**Group 2 n = 36**	**P value**
Neurosyphilis treatment: Intravenous Penicillin G/Ceftriaxone (%)	72 (75.8)	45 (76.3)	27 (75.0)	0.659
Syphilis treatment: Penicillin G Benzathine (%)	21 (22.1)	12 (20.3)	9 (25.0)	
Another option (%)	2 (2.1)	2 (3.4)	0 (0)	
**Case 5: PLHIV diagnosed recently not under HAART, CD4^+^ cell count = 510 cells / mm³ and undetectable viral load. FTA-Abs reagent and VDRL 1:128. CSF: VDRL non-reagent, FTA-Abs reagent, 22 cells/mm^3^, protein 55 mg/dl** **3**				
	**Total n = 96**	**Group 1 n = 60**	**Group 2 n = 36**	**P value**
Neurosyphilis treatment: Intravenous Penicillin G/Ceftriaxone (%)	88 (91.7)	54 (91.5)	34 (94.4)	0.706
Syphilis treatment: Penicillin G Benzathine (%)	8 (8.3)	6 (10.2)	2 (5.6)	
Another option (%)	0 (0)	0 (0)	0 (0)	

1 Missing/do not know for three participants (3.1%);

2 missing/do not know for one participant (1.0%);

3 missing/do not know for two participants (2,0%); HAART, highly active antiretroviral treatment. PLHIV = people living with human immunodeficiency virus; HAART = Highly Active Antiretroviral Therapy; FTA-Abs = fluorescent treponemal antibody absorption; VDRL = Venereal Disease Research Laboratory; CSF = cerebrospinal fluid.

** Group 1: Participants reporting no difficulties for lumbar puncture or patient hospitalization;

*** Group 2: Participants reporting at least some difficulties for lumbar puncture and/or those who perceived patient hospitalization as very difficult.

The first two vignettes described a PLHIV with early latent-stage syphilis and a VDRL titer of 1:128. When the CD4^+^ cell count was above 350 cells/mm^3^, 21.3% of respondents referred the patient for lumbar puncture; this percentage rose to 65.3% when the CD4^+^ cell count was below 350 cells/mm^3^.

The third vignette described a patient with early latent syphilis with a CD4^+^ cell count above 350 cell/mm^3^ and a VDRL titer of 1:128 with a four-fold (two dilution) decrease in the titer within 12 months after adequate treatment. According to 67.7% of the respondents, this patient should be referred for lumbar puncture.

The fourth and fifth vignettes presented a patient recently diagnosed with HIV infection, with latent syphilis of unknown duration. When the vignette described a patient with a CD4^+^ cell count of 110 cells/mm^3^ and a VDRL titer of 1:4, 51.6% of participants referred the patient for lumbar puncture. When the case presented a patient with a CD4^+^ cell count above 350 cells/mm^3^ and VDRL titer of 1:32, the 40.8% of respondents referred the patient to lumbar puncture. We found no statistically significant differences between Groups 1 and 2 in the answers to case vignettes 1-5 (Table 2).

In the five vignettes addressing the interpretation of CSF laboratory reports, we presented hypothetical patients with different CD4^+^ cell counts and chemocytological findings in CSF. For all situations, the treponemal serological test was reactive, while VDRL was non-reactive in CSF. Current Ministry of Health recommendations in Brazil do not define specific criteria for neurosyphilis treatment in PLHIV with a non-reactive VDRL in CSF, but underline pleocytosis as a common finding.^17^

The first two vignettes in this section presented PLHIVs with syphilis, VDRL titer of 1:128, elevated cell count in CSF and normal protein levels. For the case vignette with a CD4^+^ cell count above 350 cells/mm³, 75.8% of respondents indicated neurosyphilis treatment; when CD4^+^ cell count was below 350 cells/mm³, this percentage was 88.4%.

The third and fourth case vignettes presented a similar patient profile as previous cases with normal CSF cell counts and high protein levels; neurosyphilis treatment was indicated by 57.7% and 75.8% of the respondents for the vignettes with higher and lower CD4^+^ cell counts, respectively. The last case vignette addressed a PLHIV not on antiretroviral treatment with syphilis coinfection, who had elevated cell and protein counts in CSF. For this hypothetical patient, 91.7% of the respondents indicated neurosyphilis treatment, with similar percentages in Groups 1 and 2 (3). Again, we found no statistically significant differences between Groups 1 and 2 in responses to case vignettes in this section.

### Knowledge and attitudes regarding lumbar puncture criteria and syphilis clinical management

The 2018 Ministry of Health recommendations in Brazil suggest the use of lumbar puncture for neurosyphilis investigation in PLHIV with syphilis coinfection in the following situations: presence of neurological or ophthalmic symptoms, evidence of active tertiary syphilis, and after antibiotic treatment failure, independently of presumed sexual re-exposure.^17^ Only 23.5% (95% confidence interval, CI 14.9-32%) of the study participants provided correct answers according to the current recommendations. We found no statistically significant differences between participants who completed or were still in-course for infectious disease residency (21.3% versus 29.6%; P = 0.427) and physicians responding in paper or online forms (22.7% versus 26.1%; P = 0.781). The vast majority of professionals agree that PLHIV who present with syphilis treatment failure should be investigated for neurosyphilis, according to the current recommendations in Brazil.^17^ However, many respondents mistakenly indicated that CD4^+^ cell count, VDRL titers, and syphilis stage were part of the current guidelines criteria for lumbar puncture in this population (4).

**Table 4:** Responses to questions on management of syphilis in PLHIV with no neurologic symptoms

**According to current national recommendations, which asymptomatic individuals with syphilis-HIV coinfection should be referred for lumbar puncture for neurosyphilis investigation?**		
	**n (%)**	**95% CI**
All patients (%)	10 (10.2)	4.1-16.3
Patients with late/indeterminate latent syphilis (%)	26 (26.5)	17.6-35.4
Patients with reduction of VDRL < 2 dilutions 3 months after adequate treatment, or < 4 dilutions 6 months after adequate treatment (%)	78 (79.6)	71.5-87.7
Patients with CD4^+^ cell count ≤ 350 mm^3^ (%)	55 (56.1)	46.1-66.1
Patients with VDRL titers ≥ 1:16 (%)	7 (7.1)	2.0-12.3
Patients with VDRL titers ≥ 1:32 (%)	24 (24.5)	15.8-33.2
**In your opinion, which asymptomatic individuals with syphilis-HIV coinfection should be referred for CSF puncture for neurosyphilis investigation?**		
	**n (%)**	**95% CI**
All patients (%)	10 (10.2)	4.1-16.3
Patients with late/indeterminate latent syphilis (%)	29 (29.6)	20.4-38.8
Patients with reduction of VDRL < 2 dilutions 12 months after adequate treatment (%)	82 (83.7)	76.2-91.2
Individuals with CD4^+^ cell count ≤ 350mm^3^ (%)	51 (52.0)	42.0-62.1
Individuals with VDRL ≥ 1:16 (%)	10 (10.2)	4.1-16.3
Individuals with VDRL ≥ 1:32 (%)	22 (22.4)	14.0-30.8
**Which diagnostic criteria you consider for neurosyphilis in asymptomatic PLHIV?**		
	**n (%)**	**95% CI**
VDRL reagent in CSF, regardless of CSF cell/protein count (%)	87 (88.8)	82.4-95.1
Elevated CSF cell count with reagent FTA-Abs (%)	58 (59.2)	49.3-69.0
Elevated CSF cell count with reagent or non-reagent FTA-Abs (%)	36 (36.7)	27.7-46.4
Elevated CSF protein count with reagent FTA-Abs (%)	49 (50.0)	39.9-60.0
Elevated CSF protein count with reagent or non-reagent FTA-Abs (%)	37 (37.8)	28.0-47.5

PLHIV = people living with human immunodeficiency virus; HIV = human immunodeficiency virus; CI = confidence interval; VDRL = Venereal Disease Research Laboratory; CSF = cerebrospinal fluid; FTA-Abs = fluorescent treponemal antibody absorption.

Among the 23 participants with correct answers according to the current recommendations for asymptomatic neurosyphilis investigation in PVHIV, 5 (21.7%) expressed the opinion that indications for lumbar puncture should be more comprehensive, distributed as follows:

- Individuals with late/unknown duration latent syphilis, n = 1
- Individuals with CD4^+^ cell count ≤ 350 mm^3^, n = 5
- Individuals with VDRL titer ≥ 1:32, n = 2

### Participants’ perceptions about lumbar puncture criteria and syphilis clinical management

Participants’ opinions on criteria for referring asymptomatic PLHIV to lumbar puncture show that most believe lumbar puncture should be performed more often than currently recommended; 52.0% believe that CD4^+^ ≤ 350 cells/mm³ should be a criterion for lumbar puncture; 29.6% believe that patients with late latent/unknown duration stage should be referred to lumbar puncture, and 22.4% that VDRL ≥ 1:32 should be considered for lumbar puncture.

Concerning CSF interpretation for neurosyphilis diagnosis, 88.8% consider that a reactive VDRL in CSF, regardless of cell or protein content, is a sufficient criterion. For CSF results showing a non-reactive VDRL and a reactive FTA-Abs (Fluorescent treponemal antibody absorption), most participants consider elevated CSF cell count (59.2%) and elevated protein count (50.0%) as criteria for neurosyphilis diagnosis.

Regarding treatment, all respondents considered penicillin crystalline as an adequate option. Ceftriaxone was also reported as an adequate treatment option by 43.9% (95% CI 34.2-54.0%). We did not explore whether the responders considered ceftriaxone a reliable first-line treatment.

## DISCUSSION

The results of this cross-sectional study highlight heterogeneities in the knowledge and practices of 98 infectious disease specialists and infectious disease residents from São Paulo, Brazil, regarding the clinical management of neurosyphilis investigation in asymptomatic PLHIV. Most participants believe that the criteria for lumbar puncture should be extended; almost 60% believe that low CD4^+^ cell counts should be an indication, and around a third favor late latent syphilis as a criterion to proceed with lumbar puncture even in asymptomatic patients. It is interesting to note that only 23.5% provided answers in accordance with the Guideline recommendations in Brazil, Ministry of Health. This percentage did not significantly differ among those in the residency program and graduated infectious disease specialists.

Our survey pooled infectious disease consultants from three reference centers in São Paulo. In our sample population, 44% had postgraduate degrees, and more than 90% reported providing medical care to PLHIV. This sample is not representative of all clinicians taking care of patients with HIV/syphilis coinfection in Brazil. Respondents may be better updated with current guidelines and interested in the topic. In our survey, less than a quarter of the respondents provided correct answers for lumbar puncture indications in PLHIV with syphilis. Thus, it is reasonable to assume that this percentage would be even lower among non-infectious disease clinicians or among medical practitioners in rural areas.

Cabana et al. described a lack of familiarity as a reason for not following a guideline for up to 89% of physicians.^18^ We believe that heterogeneities and recent modifications regarding recommendations for lumbar puncture among PLHIV across local and international guidelines are also likely to contribute to this low percentage of correct answers. Adherence to guideline recommendations could also be influenced by environmental-related barriers.^18,27^ We hypothesized that physicians’ perceived barriers to refer patients to lumbar puncture or to in-hospital treatment could influence questionnaire responses. Almost 40% of study participants considered access to lumbar puncture somewhat difficult or very difficult and/or hospitalization very difficult. However, we failed to find statistically significant differences in the responses to case vignettes, knowledge, and attitudes when comparing Groups 1 and 2. It is plausible to assume that significant differences could emerge among infectious disease specialists in non-referent health services, where barriers for lumbar puncture and hospitalization are higher.

There are controversies about the management of PLHIV with syphilis coinfection and no neurologic symptoms. Regarding lumbar puncture indications, some recommendations consider similar lumbar puncture criteria as those used for HIV-uninfected individuals. In Brazil, the recommendations for the management of HIV (PCDT para Manejo da Infecção pelo HIV em Adultos, 2018), the management of sexually transmitted diseases (PCDT para Atenção Integral às Pessoas com Infecções Sexualmente Transmissíveis, 2020), and The Guidelines for the Prevention and Treatment of Opportunistic Infections in Adults and Adolescents with HIV from the Centers for Disease Control and Prevention all recommend lumbar puncture for neurosyphilis investigation in PLHIV when there are neurologic symptoms, tertiary syphilis, or treatment failure. All three guideline recommendations disregard VDRL titers or CD4^+^ cell counts as criteria for neurosyphilis investigation with lumbar puncture.^16,17,28^ The 2020 European guideline on the management of syphilis highlights that robust evidence is lacking, but reiterate that some experts recommend CSF assessment in asymptomatic PLHIV with late syphilis and CD4^+^ cells ≤ 350/mm^3^ and/or a serum VDRL/RPR titer > 1:32.^29^ The 2020 German guidelines on the diagnosis and treatment of neurosyphilis consider CD4^+^ cell counts, HIV treatment status, and VDRL titers in the decision for lumbar puncture among PLHIV with no neurologic symptoms.^30^

The incidence of neurosyphilis is demonstrably higher among PLHIV compared to that in the general population.^31,32^ Additionally, higher VDRL titers and lower CD4^+^ cell counts have been associated with the development of neurosyphilis in this population.^33^ In a study published in 2009, Ghanem et al. showed that using VDRL titers and CD4^+^ cell counts as criteria for lumbar puncture was associated with very high sensitivity, 100% [95% CI, 70%–100%]; however, it would have demanded the investigation with lumbar puncture for 88% of patients,^34^ representing a considerable burden to the health system. Moreover, a more frequent indication for lumbar puncture in PLHIV with syphilis coinfection with no neurologic symptoms may also encounter low acceptability by patients.

The effectiveness in implementing recommendations varies considerably, with continuous debate regarding the adequate management of HIV-syphilis coinfection and great heterogeneity among physicians. This survey reflects the dilemma in clinical practice; more than 50% of study participants believe that CD4^+^ cell counts below 350 cells/mm³ should still be a criterion for lumbar puncture; almost 30% would indicate lumbar puncture for patients with latent syphilis of unknown duration; and approximately 20% would refer PLHIV for lumbar puncture when VDRL titers are ≥ 1:32.

Besides the controversy on lumbar puncture indication, the interpretation of CSF laboratory reports is another point of debate, as there is no gold standard for neurosyphilis diagnosis. The Ministry of Health recommendations in Brazil to refer a patient for neurosyphilis treatment do not define specific thresholds for cell or protein levels in CSF when VDRL is negative. A positive VDRL in CSF in the absence of blood contamination is highly specific but lacks diagnostic sensitivity.^35^ For PLHIV, elevated CSF cell and protein levels can occur because of HIV infection, especially when CD4^+^ cell counts are higher. Some authors suggest interpretation based on CSF cell count along with CSF treponemal test results with different cutoffs, depending on the patient's immune status.^36^ The CSF protein level is neither specific nor sensitive,^37^ but it is nevertheless considered for defining neurosyphilis in many published papers^33,34,38^ since higher levels can be associated with neurosyphilis with cutoffs that vary from 45 to 50 mg/dL.^13^ In our study, elevated cell and protein levels were considered as criteria for neurosyphilis by 59% and 50% of participants, respectively, when CSF VDRL was negative and CSF treponemal was positive.

Neurosyphilis treatment was addressed in one multicenter clinical trial including 36 PLHIV with syphilis coinfection. The authors randomized participants to receive either ceftriaxone 2 g/day or Penicillin G 24 million units/day for 10 days. Only 30 patients were included in the final analysis and the study failed to find differences between groups in the proportions of subjects with improvements in CSF cell count or protein levels.^39^ Due to scarcity of data and study limitations, the evidence is insufficient to allow the adoption of ceftriaxone as a first-line treatment for neurosyphilis.^40^ In our survey, all respondents accepted penicillin G as the antibiotic of choice and only 44% indicated ceftriaxone as a reliable option.

Our study had some limitations. Only 32.7% of all eligible infectious disease clinicians working in the participating sites responded to the questionnaire, which might have resulted in selection bias. The study instrument, a self-completion survey with close-ended questions, may have facilitated participants to provide the correct answers by chance. For the online questionnaire, professionals may have consulted guidelines and other technical manuals, leading to answers that do not necessarily reflect their attitudes and knowledge. Finally, we were limited by a small sample, which included participants from referral centers in the largest city of Brazil. The inclusion of participants from other regions could have highlighted wider gaps in knowledge and potentially identified a significant impact of barriers to refer patients to lumbar puncture or to in-hospital treatment on attitudes toward lumbar puncture.

## CONCLUSIONS

This study highlights heterogeneities in the clinical management of patients with HIV-syphilis coinfection and no neurologic symptoms, despite the existence of national guidelines. Further, our results suggest that non-adherence with guideline recommendations may result from both a lack of agreement and lack of awareness. Most infectious disease specialists consider syphilis stage, VDRL titers, and CD4^+^ cell counts as important parameters when deciding which patients need a lumbar puncture for the investigation of neurosyphilis. We failed to find statistically significant differences in attitudes and practices comparing participants who reported barriers for referring patients for lumbar puncture and/or hospitalization with participants who perceived no such difficulties. Prospective studies with long-term follow-up of clinical outcomes after several lumbar puncture criteria are needed among PLHIV with syphilis.
